# Urinary Extracellular Vesicle–Associated EphA2 Predicts Intravesical Recurrence in Bladder Cancer

**DOI:** 10.1111/iju.70656

**Published:** 2026-10-13

**Authors:** Mamoru Hashimoto, Eisuke Tomiyama, Lan Inoki, Koji Miki, Takahito Nakayama, Saizo Fujimoto, Shingo Toyoda, Takashi Kikuchi, Ryo Ukekawa, Yutaka Yamamoto, Takafumi Minami, Kazuhiro Yoshimura, Norio Nonomura, Kazutoshi Fujita

**Affiliations:** ^1^ Department of Urology Kindai University Faculty of Medicine Osaka Japan; ^2^ Department of Urology Osaka University Graduate School of Medicine Osaka Japan; ^3^ FUJIFILM Wako Pure Chemical Corporation Hyogo Japan

**Keywords:** bladder cancer, EphA2, extracellular vesicles, recurrence, urinary biomarker

## Abstract

**Objective:**

To examine the clinical usefulness of EphA2 detected on urinary extracellular vesicles (uEphA2) for bladder cancer detection and for estimating the risk of intravesical recurrence after transurethral resection of bladder tumor (TURBT).

**Methods:**

An enzyme‐linked immunosorbent assay was established to measure EphA2‐positive urinary extracellular vesicles. The diagnostic cohort comprised 191 participants, including 120 patients with bladder cancer and 71 controls.

**Results:**

uEphA2 concentrations were higher in patients with bladder cancer than in controls. Receiver operating characteristic analysis yielded an area under the curve of 0.679, with 46.7% sensitivity and 87.3% specificity. Among patients evaluable for post‐TURBT recurrence, high uEphA2 levels were associated with shorter intravesical recurrence‐free survival. This association remained significant in multivariable Cox regression analysis after adjustment for the EORTC risk score and age.

**Conclusions:**

uEphA2 showed modest diagnostic accuracy but high specificity and was independently associated with intravesical recurrence. These results support its potential use as a non‐invasive urinary marker for post‐TURBT risk stratification.

**Abbreviations**
AUC: area under the curve
EORTC: European Organization for Research and Treatment of Cancer
EphA2: ephrin type‐A receptor 2
EVs: extracellular vesicles
NMIBC: non–muscle‐invasive bladder cancer
ROC: receiver operating characteristic
TURBT: transurethral resection of bladder tumor
uEphA2: urinary extracellular vesicle–associated EphA2
uEVs: urinary extracellular vesicles

## 1 Introduction

Bladder cancer is a common urological malignancy and includes a broad clinical spectrum ranging from papillary non‐muscle‐invasive tumors to muscle‐invasive disease with substantial metastatic potential [1, 2]. For patients treated with transurethral resection of bladder tumor (TURBT), accurate identification of those at risk of recurrence is important because post‐treatment management is largely determined by surveillance intensity and the need for additional intravesical therapy.

Follow‐up after TURBT still depends mainly on cystoscopy. Although cystoscopy is reliable, repeated examinations are invasive and place a considerable burden on patients and health‐care systems [3, 4]. Urine cytology is useful in selected settings, particularly for high‐grade disease, but its sensitivity is insufficient for many recurrent tumors, and interpretation can vary among observers [5]. Accordingly, urinary biomarkers that can complement cystoscopy and cytology, especially markers that provide recurrence‐related information, remain clinically desirable [3, 6].

Extracellular vesicles (EVs) are membrane‐bound particles released from living cells into body fluids. Their molecular cargo, including proteins and nucleic acids, can mirror biological features of the producing cells. In bladder cancer, urine is an accessible source of vesicles released from the tumor and urothelial cells. Proteomic analyses of urinary EVs have identified several candidate molecules associated with bladder cancer, supporting the use of uEVs as a platform for non‐invasive biomarker development [7, 8, 9].

Further proteomic analysis focused on membrane proteins upregulated in urinary EVs from patients with bladder cancer as candidate biomarkers. Among these proteins, EphA2 was the only candidate that remained significantly elevated in patients with bladder cancer compared with patients with non‐malignant hematuria, including cystitis [9]. EphA2 is a transmembrane protein and may therefore be present on the surface of EVs [9]. EphA2 is also involved in proliferation, migration, invasion, and oncogenic signaling in bladder cancer [9, 10, 11]. In our previous study, we developed an EV‐EphA2‐CD9 enzyme‐linked immunosorbent assay (ELISA) in which isolated urinary EVs were captured with an anti‐EphA2 antibody and detected with an anti‐CD9 antibody, thereby enabling measurement of EphA2 on urinary EVs [9]. In the present study, we modified the measurement procedure by directly applying undiluted urine supernatants to anti‐EphA2 antibody‐coated plates without prior EV isolation, followed by detection with an anti‐CD9 antibody. The association between uEphA2 and intravesical recurrence after TURBT remains unclear.

We therefore assessed whether uEphA2 could distinguish bladder cancer from non‐malignant conditions and whether it was associated with intravesical recurrence after TURBT.

## 2 Materials and Methods

This study used an ELISA designed to quantify EphA2‐positive urinary extracellular vesicles. Bladder cancer was confirmed histologically as urothelial carcinoma in all cases. Controls included healthy volunteers and patients with benign urological conditions. Diagnostic performance was assessed in the full cohort, and recurrence analyses were performed in patients who underwent TURBT and cystoscopic follow‐up. The study protocol was approved by the Institutional Review Board of Kindai University Hospital (Approval number: R07‐057). Written informed consent was obtained from all participants, and the study was conducted in accordance with the Declaration of Helsinki.

After TURBT, patients generally underwent cystoscopic follow‐up every 3 months. After 1–2 years, the follow‐up interval could be extended at the physician's discretion.

Pathological diagnosis was made by experienced pathologists using hematoxylin and eosin‐stained specimens. Tumor stage was assigned according to the 7th edition of the AJCC TNM classification, and tumor grade was recorded using the 2016 WHO classification. Urine cytology was interpreted according to the Paris System; “Suspicious for High‐Grade Urothelial Carcinoma,” “Low‐Grade Urothelial Carcinoma,” and “High‐Grade Urothelial Neoplasia” were treated as positive results. The EORTC recurrence risk score was calculated as previously described [12].

Urine samples from patients with bladder cancer were collected immediately before TURBT. Samples from non‐malignant controls were obtained by spontaneous voiding. After collection, urine samples were refrigerated at 4°C and processed within 6 h. Samples were centrifuged at 2000 × g for 30 min, and the resulting supernatants were stored at −80°C until measurement.

### 2.1 Enzyme‐Linked Immunosorbent Assay (ELISA)

Ninety‐six‐well ELISA plates coated with anti‐EphA2 antibody (clone 2A1) were used. Undiluted urine supernatants (100 μL per well) were applied to the wells and incubated with shaking for 2 h at room temperature. Each sample was measured in duplicate. After three washes, biotinylated anti‐human CD9 antibody (1 K, FUJIFILM Wako) was added for 1 h. The plates were washed again, followed by incubation with horseradish peroxidase‐conjugated streptavidin for 2 h at room temperature. After five washes, tetramethylbenzidine substrate was added for 30 min. The reaction was stopped with 2 M sulfuric acid, and absorbance was read at 450 nm using a microplate reader (Infinite 200 Pro, TECAN). Exosomes isolated from the COLO201 cell line (FUJIFILM Wako) were used to generate the standard curve. uEphA2 concentrations were calculated using the standard curve and expressed in ng/mL.

### 2.2 Statistics

Categorical variables were compared using the chi‐square test or Fisher's exact test, and continuous variables were compared using the Mann–Whitney U test. For comparisons involving three groups, the Kruskal‐Wallis test followed by Bonferroni correction was applied. Receiver operating characteristic (ROC) analysis for intravesical recurrence was used to determine the uEphA2 cut‐off value using EZR. Intravesical recurrence‐free survival was estimated using the Kaplan–Meier method and compared with the log‐rank test. Cox proportional hazards models were used to identify factors associated with intravesical recurrence. Statistical analyses were performed with EZR (Saitama Medical Center, Jichi Medical University, Saitama, Japan), a graphical interface for R (The R Foundation for Statistical Computing, Vienna, Austria) [13]. Statistical significance was defined as *p* < 0.05.

Intravesical recurrence‐free survival was compared between the high and low uEphA2 groups using the Kaplan–Meier method and log‐rank test.

Progression was defined as the development of muscle‐invasive or locally advanced disease, regional lymph node or distant metastasis, or bladder cancer‐specific death. Progression‐free survival was calculated from the date of TURBT to progression or the last follow‐up and was compared between the high and low uEphA2 groups using the Kaplan–Meier method and log‐rank test.

## 3 Results

The study included 191 participants: 120 patients with bladder cancer and 71 controls. The control group comprised 42 patients who underwent TURBT or bladder biopsy for a suspected bladder lesion but had no histologically confirmed urothelial carcinoma and 29 non‐malignant controls, including 18 healthy volunteers and 11 subjects with hematuria and/or urinary stones. The median age was 77 years (range, 37–101 years) in the bladder cancer group and 73 years (range, 26–87 years) in the control group. The clinical characteristics of the bladder cancer cohort are shown in Table 1. In Figure 1, benign controls, pTa tumors without CIS, and pT1/CIS or pT2 tumors were compared; all pT1 tumors and CIS cases were included in the pT1/CIS or pT2 group. uEphA2 levels differed significantly among the three groups. Compared with the benign group, uEphA2 levels were higher in both the pTa‐without‐CIS group (Kruskal‐Wallis test with Bonferroni correction, *p* = 0.003) and the pT1/CIS‐or‐pT2 group (*p* < 0.001). No significant difference was found between the pTa‐without‐CIS and pT1/CIS‐or‐pT2 groups (*p* = 1.000). Among the controls showing higher uEphA2 values in Figure 1, five had undergone TURBT or bladder biopsy for suspected bladder lesions but had no histologically confirmed urothelial carcinoma, and four had hematuria and/or urinary stones. When all bladder cancer cases were combined, uEphA2 levels were significantly higher than in non‐malignant controls. uEphA2 levels did not differ significantly between low‐ and high‐grade tumors (Mann–Whitney U test, *p* = 0.968). ROC analysis for bladder cancer detection showed an AUC of 0.679, with 46.7% sensitivity and 87.3% specificity at the optimal cut‐off value (Figure 2).

**TABLE 1:** Patient characteristics of bladder cancer (*n*, %).

Sex		
Male	96	80%
Female	24	20%
Pathological grade		
low grade	50	41.7%
high grade	70	58.3%
Pathological T stage		
Ta without CIS	60	50%
Ta with CIS	9	7.5%
T1 without CIS	29	24.2%
T1 with CIS	6	5%
CIS	6	5%
T2	10	8.3%

Abbreviation: CIS, carcinoma in situ.

**FIGURE 1:**
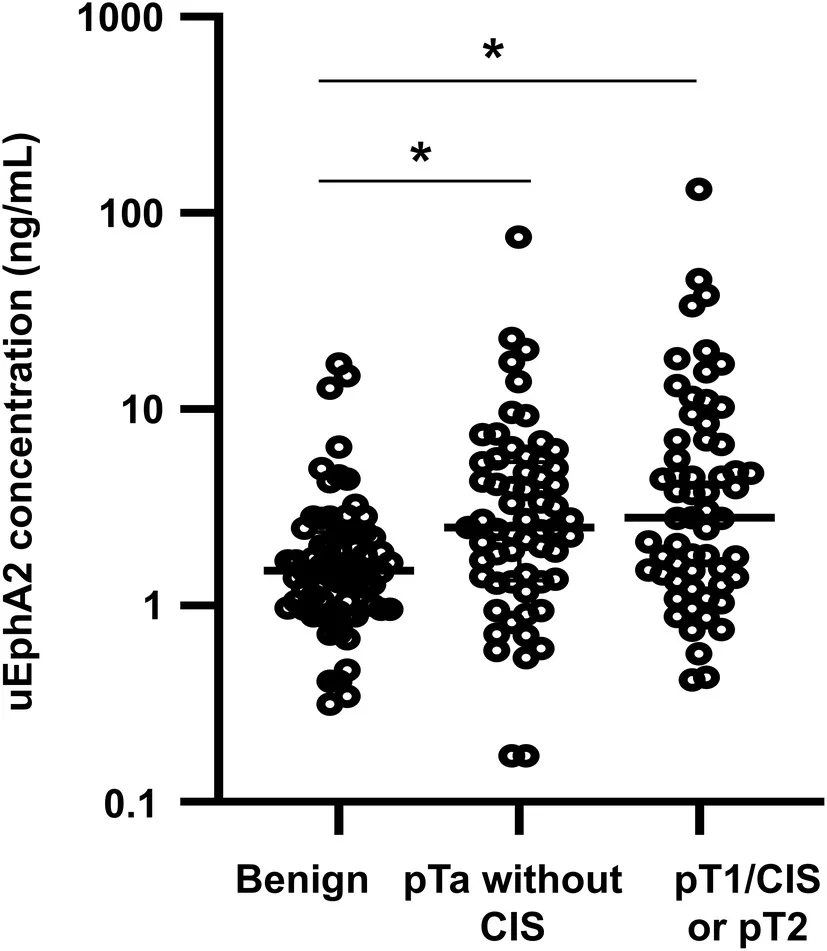
Urinary EphA2 levels in benign controls and bladder cancer patients. uEphA2 levels were compared among benign controls, pTa tumors without CIS, and pT1/CIS or pT2 tumors. Values are shown on a logarithmic scale. uEphA2 levels were significantly higher in both bladder cancer groups than in benign controls, whereas no significant difference was detected between the pTa‐without‐CIS and pT1/CIS‐or‐pT2 groups. Horizontal bars show median values. Statistical analysis was performed using the Kruskal‐Wallis test with Bonferroni correction. The pTa‐without‐CIS group comprised 60 patients. The pT1/CIS‐or‐pT2 group comprised 60 patients; all pT1 tumors and all CIS cases were included in this group. The asterisk (*) indicates a statistically significant difference (*p* < 0.05).

**FIGURE 2:**
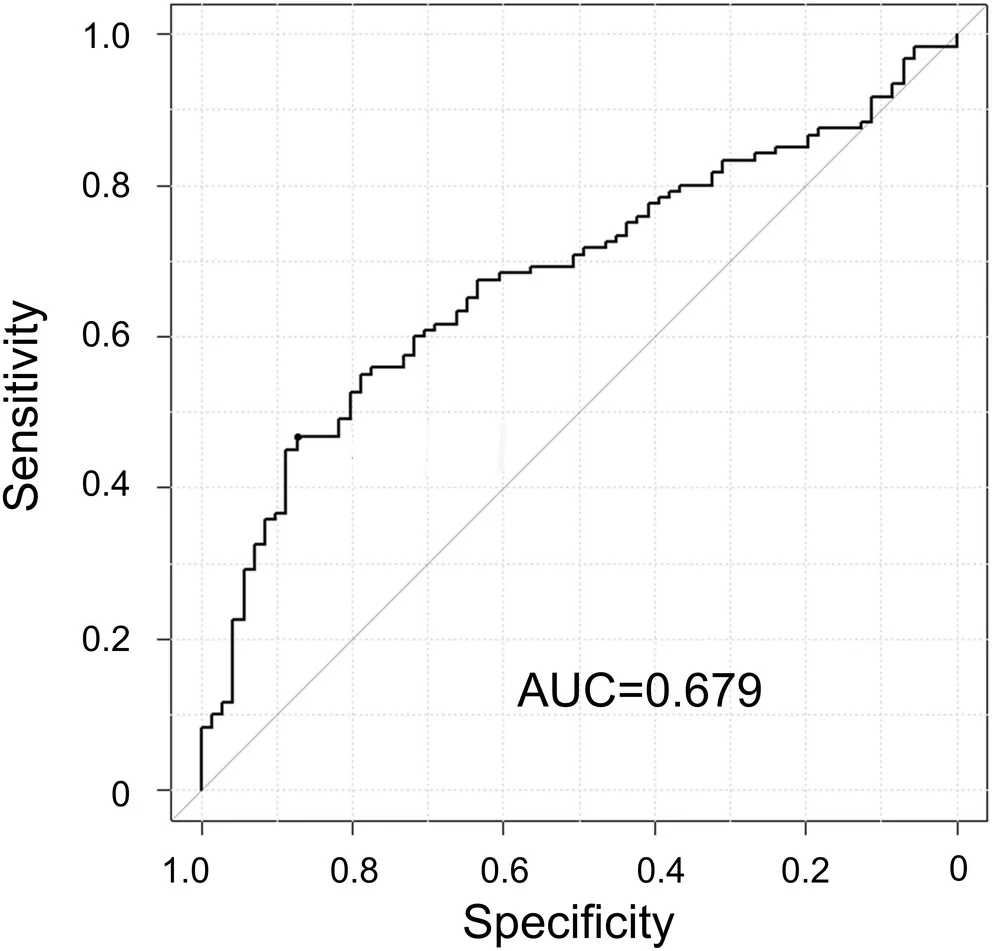
Diagnostic performance of uEphA2 for bladder cancer. Receiver operating characteristic (ROC) curve analysis was used to evaluate uEphA2 for distinguishing bladder cancer from non‐malignant conditions. The area under the ROC curve (AUC) was 0.679. At the optimal cut‐off value, sensitivity was 46.7% and specificity was 87.3%.

The pTa‐without‐CIS group comprised 60 patients. The pT1/CIS‐or‐pT2 group comprised 60 patients, including pT1 tumors without CIS (*n* = 29), pT2 tumors (*n* = 10), pTa with CIS (*n* = 9), pT1 with CIS (*n* = 6), and CIS without a papillary tumor (*n* = 6).

The recurrence analysis was restricted to patients who underwent cystoscopic follow‐up after TURBT. Patients were excluded if they received BCG treatment (*n* = 21), had pT2 disease (*n* = 10), were lost to follow‐up (*n* = 6), underwent radical cystectomy (*n* = 3), received radiation therapy (*n* = 1), or had liver metastasis (*n* = 1) or lymph node metastasis (*n* = 1). The final recurrence cohort comprised 77 patients (Table 2). Using the ROC‐derived cut‐off value of 2.047 ng/mL, patients were divided into high and low uEphA2 groups. Kaplan–Meier analysis showed significantly shorter intravesical recurrence‐free survival in the high uEphA2 group than in the low uEphA2 group (median, 8.47 months vs. not reached; log‐rank *p* = 0.044) (Figure 3).

**TABLE 2:** Patient characteristics according to uEphA2 levels (high vs. low) in bladder cancer patients followed up by cystoscopy. values are presented as *n* (%) or median (min–max).

	High uEphA2		Low uEphA2		*p*
Sex					1
Male	41	82%	22	81.5%	
Female	9	18%	5	18.5%	
Pathological grade					1
low grade	30	60%	16	59.3%	
high grade	20	40%	11	40.7%	
Pathological T stage					0.808
Ta without CIS	33	66%	22	81.5%	
Ta with CIS	5	10%	1	3.7%	
T1 without CIS	11	22%	4	14.8%	
T1 with CIS	1	2%	0	0%	
EORTC risk score					
	6	(0–13)	5	(1–10)	0.953
Recurrence					0.0327
Yes	30	60%	9	33.3%	
No	20	40%	18	66.7%	

Abbreviations: CIS, carcinoma in situ; EORTC, European Organization for Research and Treatment of Cancer; uEphA2, urinary EphA2.

**FIGURE 3:**
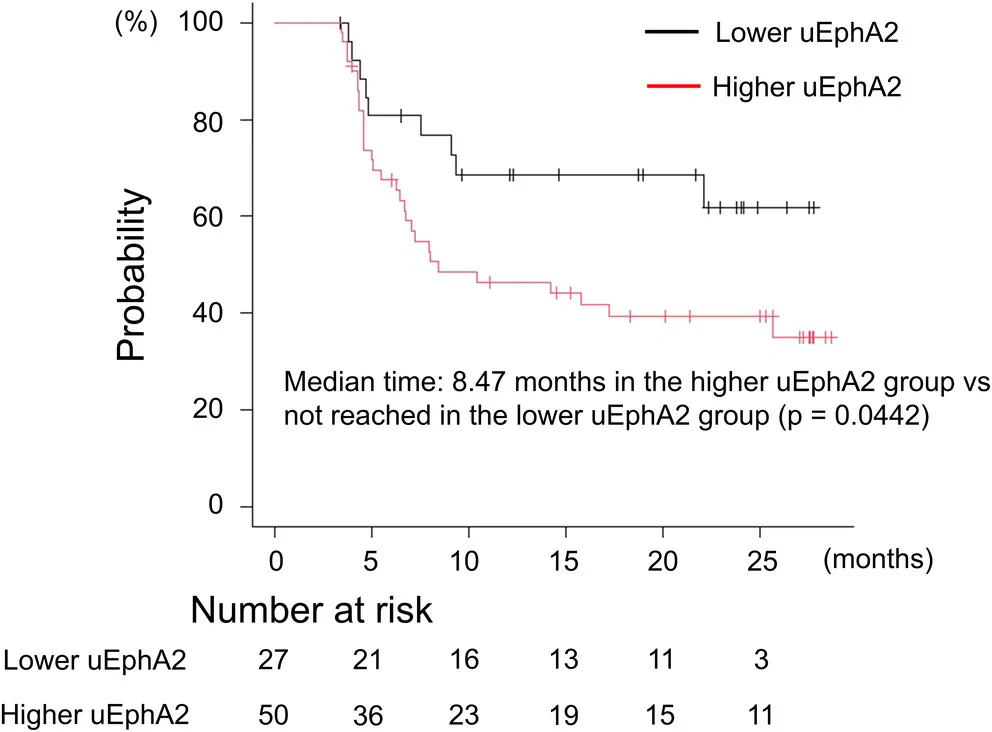
Intravesical recurrence‐free survival according to uEphA2 levels. Kaplan–Meier analysis of intravesical recurrence‐free survival after transurethral resection of bladder tumor (TURBT), stratified by uEphA2 level. Patients were classified into high and low‐uEphA2 groups using the ROC‐derived cut‐off value. The high uEphA2 group had significantly earlier intravesical recurrence than the low uEphA2 group (log‐rank test, *p* = 0.044). Median recurrence‐free survival was 8.47 months in the high uEphA2 group and was not reached in the low uEphA2 group. Tick marks indicate censored observations.

Cox proportional hazards analyses were then performed to examine variables related to intravesical recurrence (Table 3). In univariate analysis, uEphA2 above the cut‐off value was associated with a higher risk of recurrence (HR 2.11, 95% CI 1.00–4.45, *p* = 0.049). The EORTC risk score was also associated with recurrence, whereas age was not.

**TABLE 3:** Univariate and multivariate cox proportional hazards analyses for predictors of intravesical recurrence.

Univariate analysis				Multivariate analysis		
Variable	HR	95% CI	*p*	HR	95% CI	*p*
uEphA2 level (high vs. low)	2.11	1.00–4.45	0.049	2.12	1.00–4.46	0.049
EORTC risk (continuous)	1.12	1.01–1.24	0.025	1.12	1.02–1.23	0.02
Age (≥ 70 vs. < 70 years)	0.9	0.41–1.96	0.79	0.76	0.34–1.68	0.5

Abbreviations: EORTC, European Organization for Research and Treatment of Cancer; uEphA2, urinary EphA2.

In the multivariable model including the EORTC risk score and age, high uEphA2 remained significantly associated with intravesical recurrence (HR 2.12, 95% CI 1.00–4.46, *p* = 0.049). The EORTC risk score was independently associated with recurrence, but age was not a significant predictor.

Subgroup analyses were performed separately for patients with intermediate‐ and high‐risk disease based on the EORTC recurrence score categorization [14]. Within each EORTC risk group, intravesical recurrence‐free survival was compared between patients with high and low uEphA2. In the intermediate‐risk EORTC group (recurrence score 1–9; *n* = 66), recurrence occurred in 25 of 42 patients with high uEphA2 and 6 of 24 patients with low uEphA2. The high uEphA2 group had significantly shorter intravesical recurrence‐free survival than the low uEphA2 group (median, 10.43 months vs. not reached; log‐rank *p* = 0.019). In the high‐risk EORTC group (recurrence score 10–17; *n* = 10), recurrence occurred in 5 of 7 patients with high uEphA2 and 3 of 3 patients with low uEphA2. Median intravesical recurrence‐free survival was 7.93 and 9.33 months, respectively (log‐rank *p* = 0.984).

Progression occurred in 6 of 77 patients, including 4 of 50 patients with high uEphA2 and 2 of 27 patients with low uEphA2. Median progression‐free survival was not reached in either group, with no significant difference between the groups (log‐rank *p* = 0.915). Multivariable Cox analysis was not performed because of the limited number of progression events.

## 4 Discussion

This study investigated whether EphA2 measured on urinary extracellular vesicles could provide clinically relevant information in bladder cancer. The marker showed limited sensitivity but high specificity for cancer detection and, more importantly, was associated with earlier intravesical recurrence after TURBT. The association with recurrence persisted after adjustment for the EORTC risk score and age, suggesting that uEphA2 may add molecular information to conventional clinicopathological risk assessment.

The diagnostic findings should be interpreted with caution. The AUC and sensitivity indicate that uEphA2 alone is unlikely to replace cystoscopy or serve as a stand‐alone screening test. Nevertheless, the high specificity suggests that an elevated value is relatively characteristic of bladder cancer in this cohort. This profile may be useful as an adjunctive urinary test, particularly when interpreted together with cytology and clinical findings. Since available urinary markers have shown heterogeneous diagnostic performance and have not eliminated the need for cystoscopy [3, 4, 15, 16], the main value of uEphA2 may lie not simply in detection but in the biological information carried by tumor‐related vesicles.

A possible explanation for the recurrence association is that EV‐associated EphA2 reflects active tumor behavior. EVs transfer proteins and nucleic acids between cells and can capture molecular features of the cells from which they arise [7, 8, 9]. EphA2 is a receptor tyrosine kinase linked to proliferation, migration, invasion, and oncogenic signaling in urothelial carcinoma [9, 10, 11]. Therefore, urinary detection of EphA2‐bearing vesicles may represent ongoing release from biologically active tumor cells. This interpretation is consistent with the observation that uEphA2 was not clearly related to stage or grade but was associated with subsequent recurrence.

The prognostic result is clinically relevant because current NMIBC risk assessment relies mainly on clinicopathological variables. The EORTC risk score remains an established tool for estimating recurrence and progression risk [12]. In the present analysis, uEphA2 retained an association with recurrence even when the EORTC risk score was included in the model. This suggests that uEphA2 may capture a dimension of tumor biology that is not fully represented by tumor number, size, stage, grade, and prior recurrence history. If validated, this marker could help refine surveillance planning after TURBT and support more individualized decisions regarding adjuvant intravesical treatment [17, 18].

In subgroup analyses stratified by EORTC recurrence risk, the high uEphA2 group showed significantly shorter intravesical recurrence‐free survival in the intermediate‐risk group, whereas no significant difference was observed in the high‐risk group. No significant difference in progression‐free survival was observed between the high and low uEphA2 groups. These findings should be interpreted cautiously because the high‐risk subgroup included only 10 patients, and only six progression events were observed.

Several limitations should be noted. This was a single‐center study with a modest sample size, and the recurrence cohort was smaller than the diagnostic cohort. uEphA2 was measured at one preoperative time point; therefore, changes after TURBT or during surveillance were not evaluated. The multivariable model was intentionally limited because of the number of recurrence events, and residual confounding cannot be excluded. Larger prospective studies with serial urine collection are needed to confirm the independent prognostic value of uEphA2 and to determine whether adding this marker to existing risk models improves patient management. In conclusion, uEphA2 showed moderate diagnostic performance but was independently associated with intravesical recurrence after TURBT, supporting its potential role as a urinary biomarker for recurrence risk stratification in bladder cancer.

## Author Contributions

**Mamoru Hashimoto:** methodology, investigation, formal analysis, data curation, writing – original draft, writing – review and editing, visualization. **Eisuke Tomiyama:** methodology, investigation, validation, writing – review and editing. **Lan Inoki:** investigation, data curation, writing – review and editing. **Koji Miki:** investigation, data curation, writing – review and editing. **Takahito Nakayama:** investigation, data curation, writing – review and editing. **Saizo Fujimoto:** investigation, data curation, writing – review and editing. **Shingo Toyoda:** investigation, data curation, writing – review and editing. **Takashi Kikuchi:** investigation, data curation, writing – review and editing. **Ryo Ukekawa:** methodology, validation, resources, writing – review and editing. **Yutaka Yamamoto:** investigation, data curation, writing – review and editing. **Takafumi Minami:** investigation, data curation, writing – review and editing. **Kazuhiro Yoshimura:** investigation, data curation, writing – review and editing. **Norio Nonomura:** supervision, writing – review and editing. **Kazutoshi Fujita:** conceptualization, methodology, supervision, project administration, resources, writing – review and editing.

## Funding

The authors have nothing to report.

## Ethics Statement

Approval of the research protocol by an Institutional Reviewer Board: This study was approved by the Institutional Review Board of Kindai University Hospital (Approval number: R07‐057).

## Consent

Informed consent was obtained from all participants.

## Conflicts of Interest

Ryo Ukekawa is an employee of FUJIFILM Wako Pure Chemical Corporation. The other authors declare no conflicts of interest.

## Data Availability

The data that support the findings of this study are available on request from the corresponding author. The data are not publicly available due to privacy or ethical restrictions.
